# Whole-Transcriptome Sequencing of Ovary Reveals the ceRNA Regulation Network in Egg Production of Gaoyou Duck

**DOI:** 10.3390/genes15010009

**Published:** 2023-12-20

**Authors:** Lei Zhang, Rui Zhu, Guobo Sun, Jian Wang, Qisheng Zuo, Shanyuan Zhu

**Affiliations:** 1 Jiangsu Key Laboratory for High-Tech Research and Development of Veterinary Biopharmaceuticals, Jiangsu Agri-Animal Husbandry Vocational College, Taizhou 225300, China; leizhang@jsahvc.edu.cn (L.Z.); rzhu2021@jsahvc.edu.cn (R.Z.); 2007020169@jsahvc.edu.cn (G.S.); tzwjian@126.com (J.W.); 2 Key Laboratory of Animal Breeding Reproduction and Molecular Design for Jiangsu Province, College of Animal Science and Technology, Yangzhou University, Yangzhou 225009, China; 006664@yzu.edu.cn

**Keywords:** Gaoyou duck, egg performance, whole transcriptome, ceRNA regulatory network, double-yolked egg

## Abstract

To investigate the regulatory mechanism of the competing endogenous RNAs (ceRNAs) on the egg performance of Gaoyou ducks, full transcriptome sequencing was performed to analyze the ovarian tissues in Gaoyou ducks. The ducks were categorized into high- and low-yield groups based on the individual in-cage egg production records and the hematoxylin–eosin (HE) staining results. The differentially expressed genes (DEGs), long non-coding RNAs (lncRNAs), and circular RNAs (circRNAs) were further processed by GO (gene ontology) and KEGG (Kyoto Encyclopedia of Genes and Genomes) analyses. In total, 72 DEmRNAs; 23 DElncRNAs; 4 DEcircRNAs; and 5 signaling pathways, including the ovarian steroidogenesis, PI3K-Akt, hedgehog, tryptophan metabolism, and oocyte meiosis signaling pathways, were significantly enriched. These results suggest that they could be associated with the Gaoyou duck’s ovarian function and affect the total egg production or double-yolked egg production. Furthermore, a coregulation network based on the related candidate ceRNAs across the high- and low-yield egg production groups was constructed. Our findings provide new insights into the mechanisms underlying the molecular regulation of related circRNA/lncRNA–miRNA–mRNA in the egg production and double-yolked egg traits of Gaoyou ducks.

## 1. Introduction

The Gaoyou duck is a type of Shelduck that is reared for both eggs and meat. It is one of the three most popular duck breeds in China, originating from the Gaoyou region of Jiangsu Province. This duck species has been raised in the Jianghuai region for hundreds of years and is well-known both domestically and internationally for its ability to produce double-yolked eggs [1]. The double-yolked eggs represent a cultural moral of “good luck and happiness”, and the Gaoyou duck has gained popularity as a local breed following a long selection and breeding process. The percentage of double-yolked eggs in Gaoyou ducks ranges from 2 to 10% [2]. The double-yolked trait of the Gaoyou duck is an embodiment of fitness and it is an excellent material with which to study the egg production mechanism of double yellow eggs. However, little progress has been made regarding the mechanism underlying the egg production performance and the double-yolked egg trait of Gaoyou ducks, which limits the development and utilization of this breed.

The egg production performance of poultry mainly depends on the development of follicles on the ovaries, which is a highly coordinated and complex physiological process [3]. Upon reaching sexual maturity, the primordial follicles in the ovaries grow gradually towards the hierarchical follicles and finally grow into a dominant follicle [4]. The ovum is then released to enter the fallopian tubes and ultimately forms an egg [5]. Double-yolked eggs are formed by the simultaneous development and ovulation of two follicles on the ovaries of poultry, which are accepted by the fallopian tubes at the same time [6]. Generally, the double-yolked egg rate in poultry is extremely low. Although double-yolked eggs have higher nutritional value than single-yolked eggs, they are considered a type of deformed egg in the breeding process and usually do not have the ability to hatch, resulting in relatively lagging research [7]. Salamon et al. [8] have demonstrated that when the size and position of double-yolked eggs are appropriate, healthy poultry offspring can be hatched, breaking the traditional view that double-yolked eggs cannot be hatched and revealing the possibility of stable inheritance of double-yolked egg performance. Furthermore, Liu et al. [9] explored the double-yolked egg trait of Zhedong white goose, and they discovered that the high level of IGF1 in plasma could cause the production of double-yolked eggs. However, the production of double-yolked eggs [10] is governed by a complex regulation mechanism, and there is more than one factor, causing it to remain obscure. 

Transcriptome analyses of ovarian tissues have revealed several genes that may be involved in the regulation of egg production in chickens, as demonstrated by earlier research [11,12,13]. By comparing the transcriptomes of three stages of ovarian follicles in chicken groups with and without better egg production, Sun et al. [12] found that two candidate genes—*NDUFAB1* and *GABRA1*—participate in the egg-laying performance of Jilin Black chickens. Mishra et al. [13] explored the RNA-Seq data from the hypothalamic–pituitary–ovarian axis of Luhua chickens with high- and low-egg production and found that four potential signaling pathways (the mTOR, Jak-STAT, Tryptophan metabolism, and PI3K-Akt signaling pathways) might be involved in the reproductive biology of Luhua chickens. The reported research on the properties of double-yolked eggs is comparatively limited to certain gene or protein levels, and little research has been conducted on the regulation of gene expression through networks based on whole-transcriptome RNA-Seq that is mediated by non-coding RNA or ceRNA (the competing endogenous RNA). Considering the special egg production capacity of Gaoyou ducks, the number of single-yolk eggs and double-yolk eggs should be intensively explored through pivotal mRNAs, lncRNAs (long non-coding RNAs), and circRNAs (circular RNAs) for a better understanding of its comprehensive regulation mechanism of the double-yolked egg trait. To explore the mechanism of egg production performance of Gaoyou ducks, especially for its double-yolked egg trait, this study combined individual cage-rearing records and RNA-Seq methods to screen high- and low-yield egg production groups of Gaoyou ducks. A full transcriptome sequencing analysis was performed on their ovaries to screen for DEmRNAs, DElncRNAs, and DEcircRNAs, thus constructing a ceRNA regulatory network related to the egg production performance trait of Gaoyou ducks. Our findings provide new insights into the mechanisms underlying the molecular regulation of a related ceRNA network of circRNA/lncRNA–miRNA–mRNA in the egg production and double-yolked egg trait of Gaoyou ducks. 

## 2. Materials and Methods

### 2.1. Sample Collection 

Individually housed in distinct single cages, 80 female Gaoyou ducks were raised at the National Gene Bank of Waterfowl Resources in Jiangsu, China, maintaining uniform environmental and nutritional conditions until reaching 330 days of age. The ducks were divided into a high-yield group and a low-yield group based on individual egg production data (68 valid individual records). Subsequently, three individuals were selected from the high-yield group (sample numbers: #20, #29, and #35, Group G) and the low-yield group (sample numbers: #22, #23, and #24, Group D), and the ducks were sacrificed by exsanguination. After the removal of egg yolks, ovarian stroma samples were collected and promptly snap-frozen in liquid nitrogen in −196 °C for subsequent experiments.

### 2.2. RNA Isolation, Library Construction, and Sequencing

According to the manufacturer’s instructions, total RNA was extracted with Trizol regent (Ambion, Austin, TX, USA) from the ovarian tissue of the Gaoyou ducks. The quantity and quality of total RNA was evaluated using a NanoDrop ND1000 (Thermo Scientific, Waltham, DE, USA) and an Agilent 2100 Bio-analyzer (Agilent Technologies, Palo Alto, CA, USA). Qualified RNA samples were subsequently transported to OE Biotech CO., LTD in Shanghai, China, for comprehensive whole-transcriptome sequencing. TruSeq Stranded Total RNA with Ribo-Zero Gold reagent (Illumina, CA, USA) was used to remove rRNA. Utilizing the Illumina HiSeq2500 system sequencing platform, 6 libraries, which underwent the sequencing process, were generated as described [12]. 

### 2.3. Transcriptome Data Analysis

The clean reads were obtained from the raw reads using Trimmomatic software (Version 0.33) [14]. Then, the clean reads were mapped to the reference genome (BGI duck 1.5 reference) using hisat2 (Version 2.2.0) for the downstream analysis, as previously described [15]. By using DESeq (Version 2.10), the gene counts of each sample were standardized, thus to calculate the multiple times of differential genes. mRNAs, lncRNAs, and circRNAs with an absolute log_2_|fold-change| ≥ 2 and an adjusted *p*-value of ≤0.05 were differentially expressed mRNAs, lncRNAs, and circRNAs in this study [16,17,18,19]. The functions of mRNA, lncRNA, and circRNA were individually identified using the gene ontology (GO) database. For the classification of unigenes, Kyoto Encyclopedia of Genes and Genomes (KEGG) pathways were analyzed through the online KEGG Automatic Annotation Server. The raw sequencing data were deposited in the Sequence Read Archive at NCBI under the accession number PRJNA1033811. 

### 2.4. Hematoxylin–Eosin (HE) Staining and Analysis of Ovarian Follicles

The ovarian tissues of #51 and #43 Gaoyou ducks were separated and compared as representative individuals between the high-yield group and low-yield group, and the development of the ovarian follicles was compared using a microscope. Following the categorization by follicle diameter, various grades of follicles were distinguished, including small white follicles (SWF), large white follicles (LWF), and small yellow follicles (SYF), as described by Kun Zou et al. [20]. The ovarian tissues were rinsed in PBS solution and quickly placed in 4% formaldehyde fixative. After fixation, washing, dehydration, clearing, wax dipping and embedding, sectioning, spreading, sticking, and sealing, the samples were dried and stained. Subsequently, the ovarian follicle sections were observed with an optical microscope. The amounts of SWF, LWF, and SYE were counted in three sections in each individual Gaoyou duck. 

### 2.5. Analysis through Quantitative Reverse Transcription Polymerase Chain Reaction (qRT-PCR) 

For the validation of the whole-transcriptome sequencing results, six randomly chosen candidates from DEmRNAs, DElncRNAs, and DECircRNAs underwent qRT-PCR validation. Total RNA extraction from the ovary samples of Gaoyou ducks utilized the TRIzol Kit (Invitrogen). qPCR was executed on an ABI 7500 Real-time Detection System (Applied Biosystems). The quantification of relative gene expression levels was based on the *GADPH* (glyceraldehyde 3-phosphate dehydrogenase) gene expression using the 2^−ΔΔCt^ method. The primes are supplied in Supplementary Table S1.

### 2.6. Data Analysis

Statistical analyses for this study were executed using SPSS, version 19.0 (IBM, Armonk, NY, USA). The results were presented as mean ± standard deviation, and differences were evaluated through the independent-sample *t*-test. A *p*-value < 0.05 was statistically significant. Charts were prepared in GraphPad Prism Version 6.0.

## 3. Results

### 3.1. The Identification of Individuals with High- and Low-Egg Production

**Figure 1:**
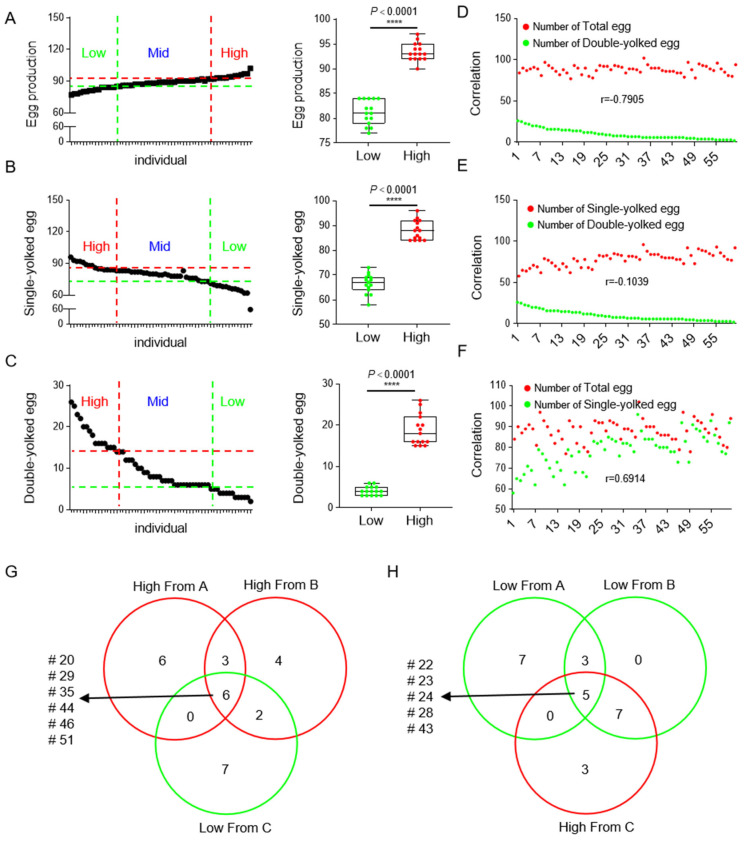
Records and analysis of individual egg production data of caged Gaoyou ducks. (**A**) Left: the total egg production records of 68 valid Gaoyou ducks; right: comparative analysis of high-egg-production individuals (top 25%) and low-egg-production individuals (bottom 25%). (**B**) Left: the total single-yolk egg production records of 68 valid Gaoyou ducks; right: comparative analysis of high single-yolk-egg-production individuals (top 25%) and single-yolk-egg-production individuals (bottom 25%). (**C**) Left: the total double-yolk egg production records of 68 valid Gaoyou ducks; right: comparative analysis of high double-yolk egg production individuals (top 25%) and low double-yolk egg production individuals (bottom 25%). (**D**–**F**) Correction analysis between total eggs, double-yolk eggs, and single-yolk eggs. (**G,H**) High-yield individuals with total egg production and single-yolk egg production both in the top 25% and double-yolk egg production in the bottom 25%; low-yield individuals with total egg production and single-yolk egg production both in the bottom 25% and double-yolk egg production in the top 25%. **** represents the *p*-value < 0.0001.

In order to accurately determine the high- and low-egg production individuals in the Gaoyou duck population, the total egg production of 68 valid Gaoyou ducks caged in the same batch was counted over a period of 4 months. Initially, the top 25% of individuals were identified as high-egg-production individuals, and the last 25% of individuals as low-egg-production individuals (Figure 1A). A significant difference in the total egg production was observed between the two groups (Figure 1D). Considering the double-yolk egg characteristic of Gaoyou ducks, the numbers of single-yolk eggs (Figure 1B) and double-yolk eggs (Figure 1C) were counted separately from the total egg production. In addition, high-double-yolk and low-double-yolk individuals were assessed using the same criteria. Notably, a significant, positive correlation was found between the data on the single-yolk eggs and the total number of eggs (Figure 1F), whereas the double-yolk egg data showed a significant negative correlation with both the number of single-yolk eggs and the total number of eggs (Figure 1E). These results indicate that, although the double-yolk egg trait is characteristic of Gaoyou ducks, it may not be beneficial for high egg production. Subsequently, Wayne’s comprehensive analysis was performed to determine individuals concurrently belonging to the top 25% in total egg production and single-yolk egg production, as well as the bottom 25% in double-yolk egg production. These individuals were defined as high-yield Gaoyou ducks, numbered #20, #29, #35, #44, #46, and #51 (Figure 1G). In contrast, low-yield individuals were defined as those belonging to the bottom 25% in terms of total egg production and single-yolk egg production, and the top 25% in double-yolk egg production, which were numbered #22, #23, #24, #28, and #43 (Figure 1H).

### 3.2. The Strategy of Sample Acquisition Ensures the Availability of RNA-Seq

**Figure 2:**
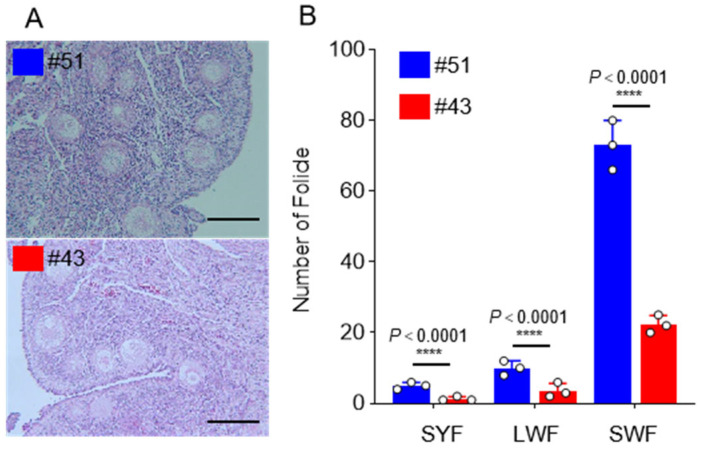
The follicular development of high- and low-yield Gaoyou ducks. (**A**) The HE staining slices of high- and low-yield Gaoyou ducks. (**B**) The follicular number statistics of high- and low-yield Gaoyou ducks. **** represents the *p*-value < 0.0001.

In order to further clarify the individual differences between the high-yield and low-yield Gaoyou ducks, the ovaries of individuals #51 and #43 were sliced and stained. The number of follicles was statistically analyzed at three levels, including small yellow follicles, large white follicles, and small white follicles (Figure 2). The results showed a higher number of follicles at all three levels in samples from #51 compared to #43. These results were consistent with the differences in individual egg production records between the high- and low-yield groups. Based on the above results, #20, #29, and #35 were selected as representatives of the high-yield Gaoyou duck group, and #22, #23, and #24 as representatives of the low-yield Gaoyou duck group. Thereafter, whole-transcriptome sequencing samples were collected from ovarian stroma tissues to screen to characterize differential genes that regulated follicular development and selection, as well as the double-yolk egg trait in Gaoyou ducks.

### 3.3. Whole-Transcriptome Library Construction and Clean Data Statistics

Six whole-transcriptome libraries were constructed from ovarian tissues of high- and low-yield Gaoyou ducks. The results are presented in Supplementary Table S2. The raw and clean reads for each library surpassed 99 million. Quality control and filtering were performed on the data, and high-quality clean reads were obtained by removing the adaptor reads and low-quality reads. The Q30 was greater than 95%, and the GC content was greater than 45%. The clean reads were aligned to the reference genome (Anas platyrhynchos), achieving a total mapping rate ranging from 89.64% to 90.15%. The analysis of sequencing data indicated reliable data quality, substantiating its suitability for subsequent analyses.

### 3.4. Analysis of Differentially Expressed Genes among Individuals with Different Egg Production

**Figure 3:**
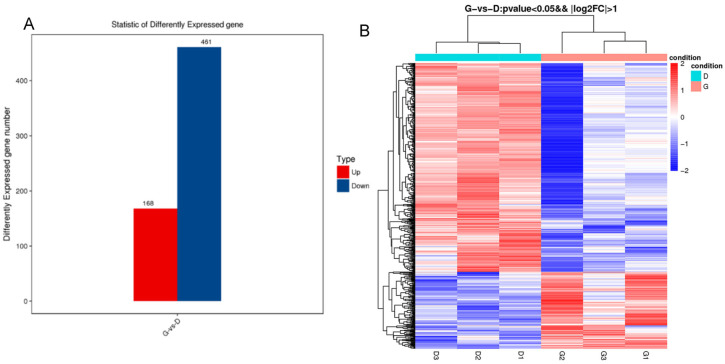
Differentially expressed genes in the ovary between high- and low-yield Gaoyou ducks. (**A**) A total of 629 genes were differentially expressed. (**B**) Cluster analysis results of different groups.

From the six ovarian tissue samples utilized for whole-transcriptome sequencing, statistical analysis of the expression results was carried out through pairwise comparisons. Figure 3A illustrates a total of 629 differentially expressed genes (DEGs), encompassing 138 upregulated genes and 461 downregulated genes. Unsupervised hierarchical clustering was performed on the differentially expressed genes, with the results showing that individuals #20, #29, and #35 were significantly clustered, while individuals #22, #23, and #24 were clustered into another group (Figure 3B). From these differential genes, *HSD11B2* [21] was involved in steroid synthesis and metabolism, and its expression in the high-yielding group was significantly higher than that in the low-yielding group. Moreover, some DEGs showed high expression in the low-yield group, including *PRL* [22], which regulates ovarian follicular development, and *DAZL* [23], which is involved in meiosis. These results are consistent with the basic characteristics of the high-yield Gaoyou duck group.

**Figure 4:**
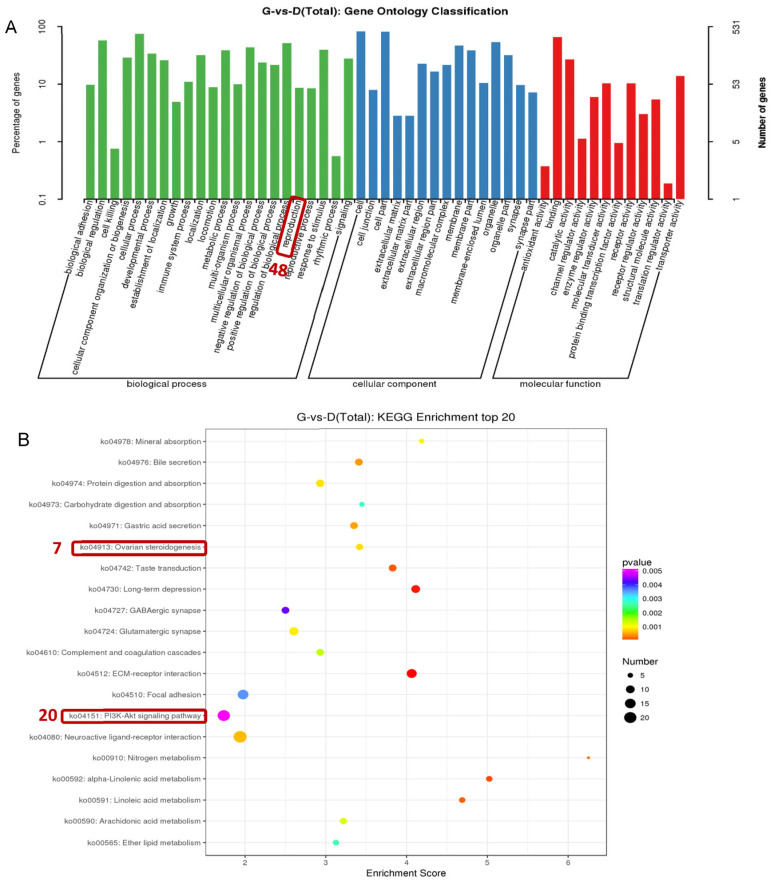
Go and KEGG analysis based on differentially expressed genes between high- and low-yield Gaoyou ducks. (**A**) Go classification based on differentially expressed genes between high- and low-yield Gaoyou ducks. (**B**) Top 20 KEGG analysis based on differentially expressed genes between high- and low-yield Gaoyou ducks.

In order to further analyze the differences in ovarian transcription between high-yielding and low-yielding individuals, GO functional annotation was performed on 629 DEGs. The results showed that 48 DEGs were enriched in GO entries related to reproduction (Figure 4A). Further differential gene KEGG signaling pathway results showed that, among the common ECM–receiver interaction, the neuroactive light receiver interaction, focal adhesion signaling pathways, etc., the ovarian steroidogenesis signaling pathway was enriched with 7 DEGs (Figure 4B). Importantly, the literature suggests that the PI3K-Akt signaling pathway is crucial for follicular development and selection [24], and our data also show that the regulation of the PI3K-Akt signaling pathway’s signal was significantly enriched (20 DEGs). Therefore, the 72 genes obtained during GO analysis and KEGG analysis were defined as candidate genes (three duplicate DEGs) regulating the high-yield egg trait in Gaoyou ducks (Figure 4). 

### 3.5. Analysis of lncRNA’s Differential Expression among Individuals with Different Egg Production

**Figure 5:**
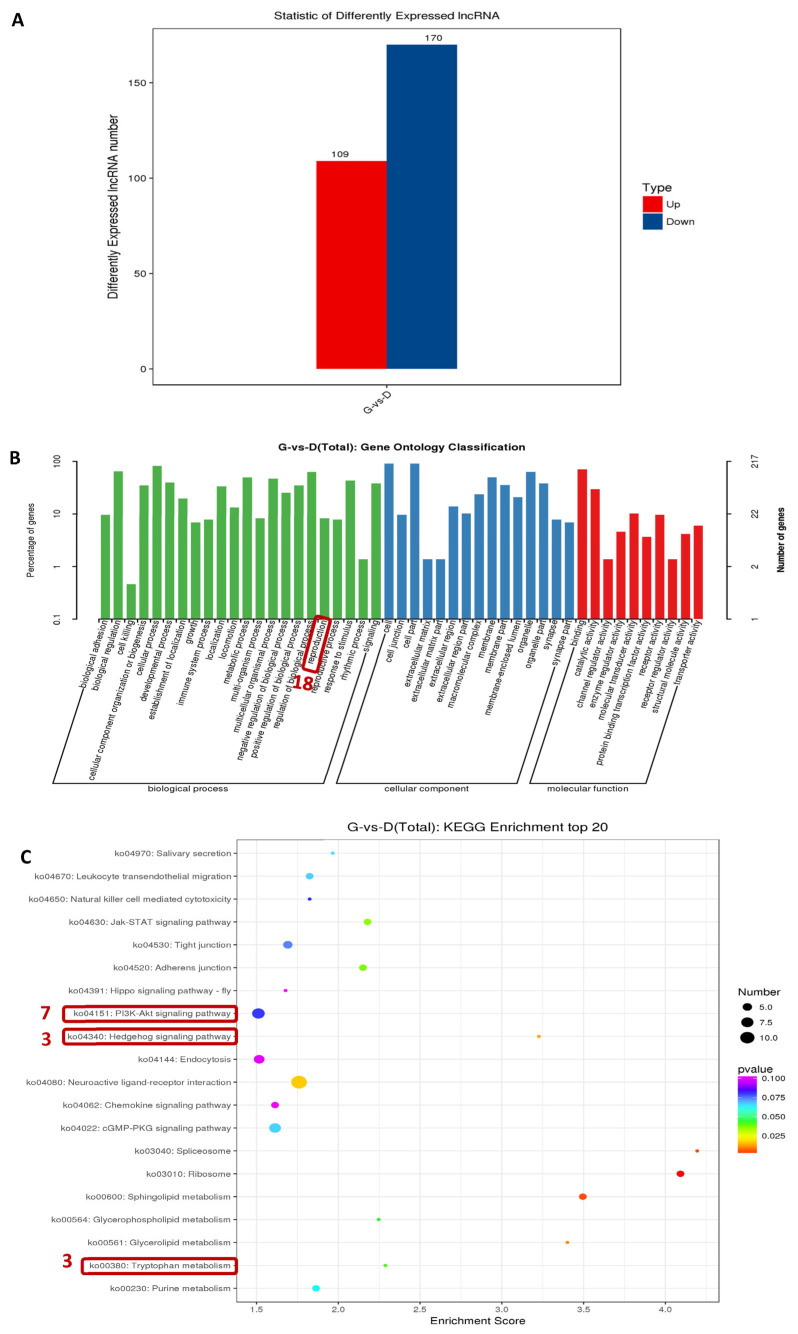
Analysis of DElncRNAs in the ovary between high- and low-yield Gaoyou ducks. (**A**) A total of 279 lncRNAs were differentially expressed. (**B**) GO classification based on DElncRNAs between high- and low-yield Gaoyou ducks. (**C**) Top 20 KEGG analysis based on DElncRNAs between high- and low-yield Gaoyou ducks.

**Figure 6:**
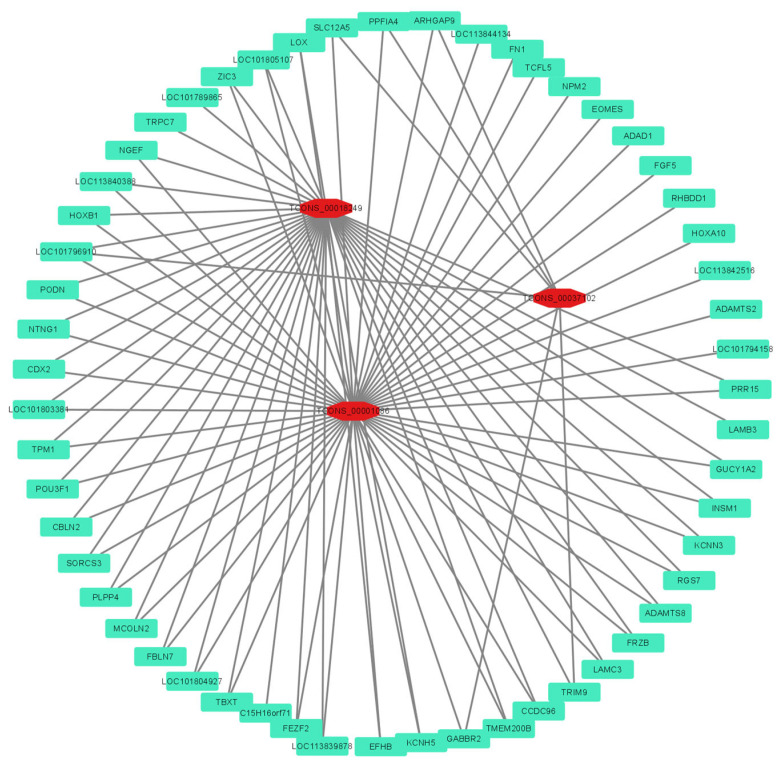
Analysis of top DElncRNA (TCONS_00037102, TCONS_00001086, and TCONS_00018249)–DEG network in the ovary between high- and low-yield Gaoyou ducks. The red circle and green square nodes represent co-differentially expressed lncRNAs and mRNAs, respectively.

In total, 279 differentially expressed lncRNAs (DElncRNAs) with an absolute log_2_|fold-change| ≥ 2 and adjusted *p*-value of ≤0.05 were screened in this study, among which 109 lncRNAs were upregulated and 170 lncRNAs were downregulated (Figure 5A). To identify the lncRNAs involved in the egg production trait of Gaoyou ducks, GO enrichment analysis was performed. As shown in Figure 5B, the specific GO term Reproduction was focused on, showing a total of 18 enriched DElncRNAs. Further KEGG signaling pathway analysis of the DElncRNAs (Figure 5C) showed that the Top 20 KEGG pathways were involved in PI3K-Akt signaling (7 DElncRNAs enriched), the hedgehog signaling pathway (3 DElncRNAs enriched), and tryptophan metabolism (3 DElncRNAs enriched). Relevant studies suggest that the hedgehog signaling pathway signaling is a key signaling pathway for follicular development and selection [25], and tryptophan metabolism is closely related to egg production performance in poultry [26]. Therefore, the 23 DElncRNAs obtained through GO analysis and KEGG analysis were identified as candidate DElncRNAs (8 duplicate DElncRNA) for regulating the high-yield egg production trait in Gaoyou ducks. To investigate the molecular regulatory networks of these 23 DElncRNAs and 72 DEGs in egg production, a network was established using the co-expressed mRNAs with a Pearson’s correlation coefficient of ≥0.8 and adjusted *p*-value of ≤0.05. Figure 6 shows the regulatory mRNA co-expression networks of *TCONS_00037102*, *TCONS_00001086*, and *TCONS_00018249*. The three DElncRNAs targeting 55 DEGs and 39 DEGs were repeatedly targeted by two of them.

### 3.6. Analysis of circRNA Differential Expression among Individuals with Different Egg Production Characteristics

**Figure 7:**
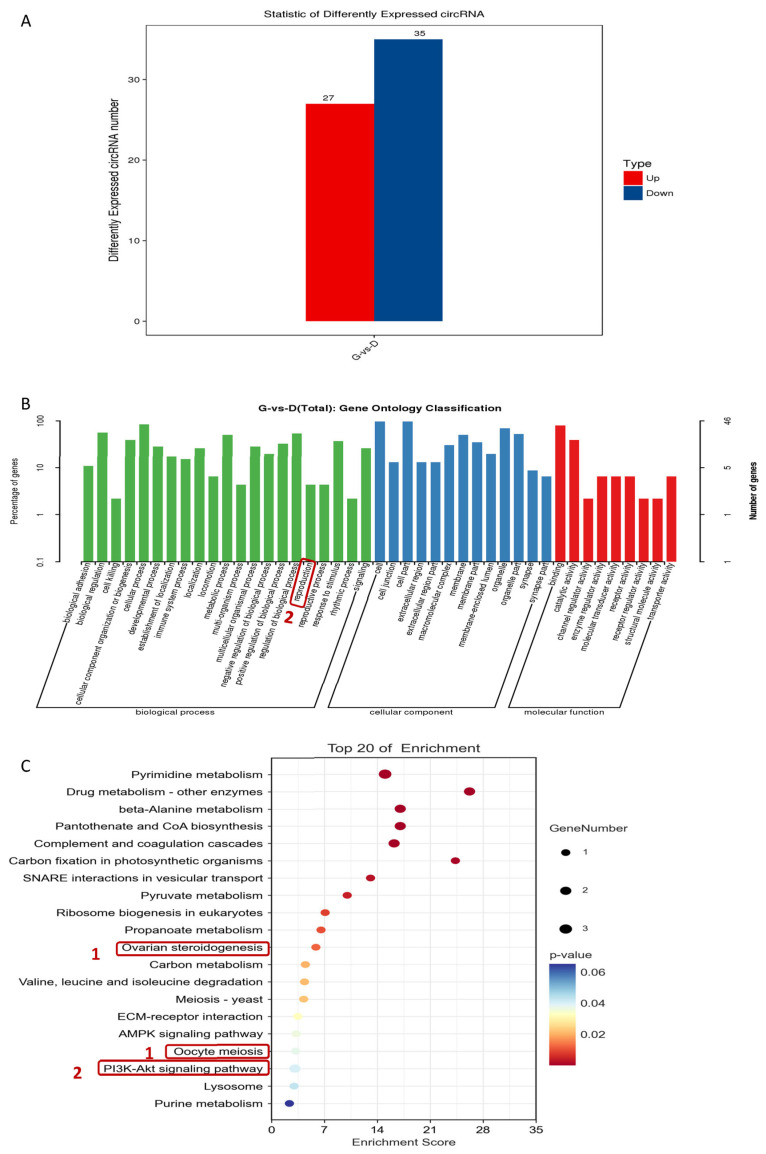
Analysis of differentially expressed circRNAs in the ovaries of high- and low-yield Gaoyou ducks. (**A**) A total of 62 circRNAs were differentially expressed. (**B**) GO classification based on differentially expressed circRNAs between high- and low-yield Gaoyou ducks. (**C**) Top 20 KEGG analysis based on differentially expressed circRNAs between high- and low-yield Gaoyou ducks.

Furthermore, circRNA prediction analysis was conducted using the whole-transcriptional data on the ovaries of the high- and low-yield groups of Gaoyou ducks. A total of 62 circRNAs were screened, with an absolute log_2_|fold-change| ≥ 2 and adjusted *p*-value of ≤0.05, among which 27 circRNAs were upregulated and 35 circRNAs were downregulated (Figure 7A). To further identify the differentially expressed circRNAs (DEcircRNAs), GO enrichment analysis was performed. As shown in Figure 7B, this study focused on the specific GO term reproduction, with 2 enriched DEcircRNAs. Further enrichment of DEcircRNAs KEGG signaling pathway results showed that (Figure 7C) the top 20 KEGG pathways were involved in ovarian steroidogenesis (1 DEcircRNA enriched), oocyte meiosis (1 DEcircRNA enriched), and the PI3K-Akt signaling pathway (2 DEcircRNAs enriched). Therefore, the four DEcircRNAs obtained via GO analysis and KEGG analysis were defined as candidate DEcircRNAs for regulating the high-yield egg production trait in Gaoyou ducks. 

**Figure 8:**
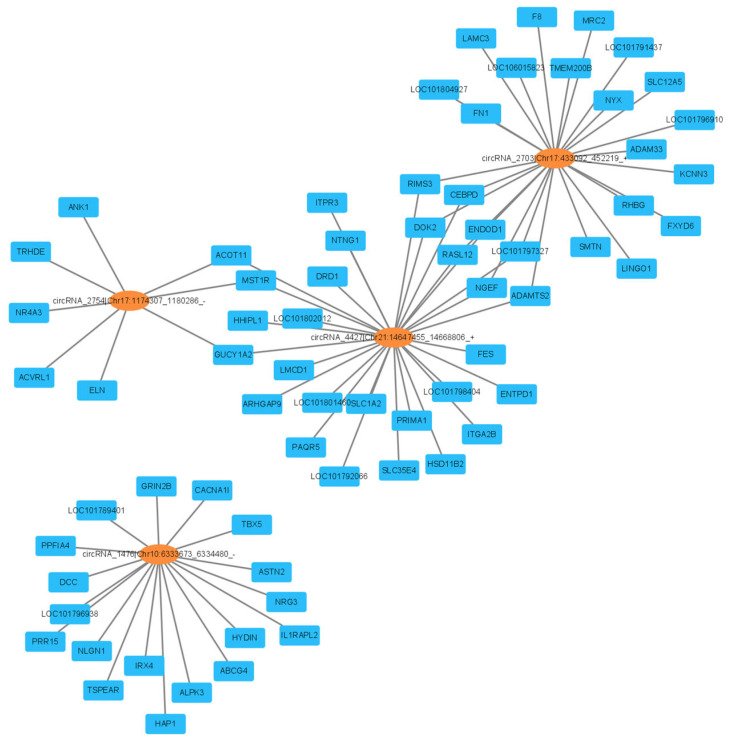
Analysis of top DEcircRNA (*circRNA_2754*, *circRNA_4427*, *circRNA_2703*, and *circRNA_1476*)–DEG network in the ovary between high- and low-yield Gaoyou ducks. The orange circle and blue square nodes represent co-differentially expressed circRNAs and mRNAs, respectively.

To investigate the molecular regulatory networks of these 4 DEcircRNAs and 72 DEGs (from Section 3.4) in the egg production of Gaoyou ducks, a circRNA-DEGs co-expression network was established with a Pearson’s correlation coefficient of ≥0.8 and an adjusted *p*-value of ≤0.05. Figure 8 shows the circRNA-DEG co-expression networks of *TCONS_00037102*, *TCONS_00001086*, *TCONS_00018249*, and *circRNA_1476*. The four DElncRNAs targeting 55 DEGs and 39 DEGs were repeatedly targeted by two of the following: *TCONS_00037102*, *TCONS_00001086*, and *TCONS_00018249*.

### 3.7. Construction of ceRNA Regulatory Network for Laying Traits in Gaoyou Ducks

**Figure 9:**
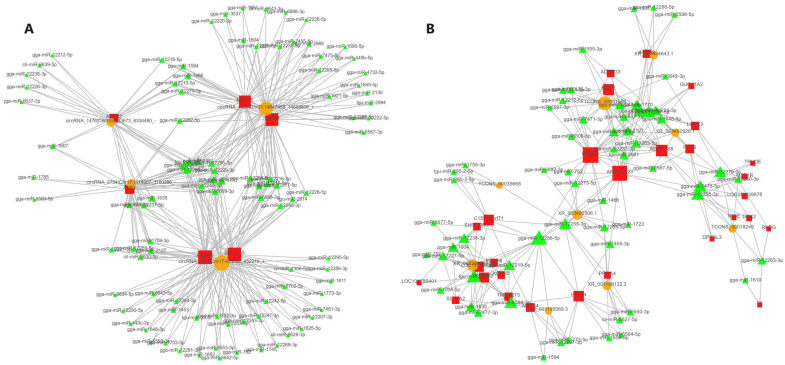
The ceRNA network, including DEmRNAs, DElncRNAs, DEcircRNAs, and miRNAs in the ovaries of high- and low-yield Gaoyou ducks. (**A**) mRNA–miRNA–lncRNA ceRNA network. The orange circle, red square, and green arrow represent co-differentially expressed lncRNAs, miRNAs, and mRNAs, respectively. (**B**) mRNA–miRNA–circRNA ceRNA network. The orange circle, red square, and green arrow represent co-differentially expressed circRNAs, mRNAs, and miRNAs, respectively.

**Figure 10:**
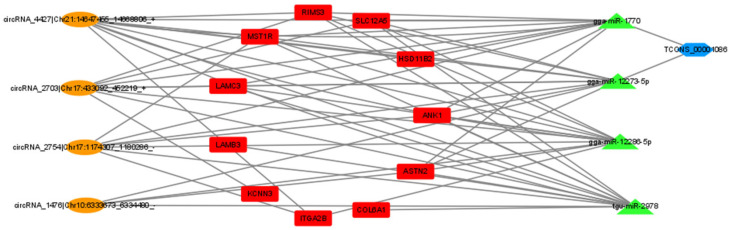
The candidate ceRNA coregulation network related to high- and low-yield egg production traits of the Gaoyou duck. The orange circle, red square, green arrow, and blue octagon nodes represent co-differentially expressed circRNAs, mRNAs, miRNAs, and lncRNAs, respectively.

Subsequently, lncRNAs were compared to miRBase using Blast [27] to identify potential miRNA precursors, and Miranda [28] software was used to predict multiple miRNA binding sites contained in circRNA. lncRNAs with miRNA precursor alignment coverage greater than 90% were selected. Considering the regulatory relationship between DElncRNA-DEmRNA and DEcircRNA-DEmRNA, we screened lncRNA, mRNA, and circRNA that were significantly differentially expressed and regulated by miRNA. The criteria for selection included a correlation coefficient of ≥0.8 and an adjusted *p*-value of ≤0.05. In total, 264 lncRNA–miRNA–mRNA interactions and 346 circRNA--miRNA-mRNA were finally obtained between the high- and low-yield Gaoyou ducks (Figure 9). To further delineate the candidate ceRNA transcript interaction relationships, 11 genes that were significantly enriched were extracted based on the above analysis, including *COL6A1*, *LAMB3*, *ITGA2B*, *LAMC3*, *HSD11BW*, *KCNN3*, *RIMS3*, *MST1R*, *ASTN2*, *SLC12A5*, and *ANK1*. To further explore the candidate ceRNA subnetwork participating in the egg production trait of Gaoyou ducks, four miRNAs were found to be shared by the high- and low-yield Gaoyou ducks’ ovaries, and another candidate DEcircRNA, DElncRNA, was extracted to construct the ceRNA regulatory network. Ultimately, a ceRNA network model was established, encompassing four miRNAs (*gga-miR-12273-5p*, *gga-miR-12286-5p*, *gga-miR-1770*, and *tgu-miR-2978*), one lncRNA (*TCONS_00001086*), four circRNAs (*circRNA_1476*, *circRNA_2703*, *circRNA_2754* and *circRNA_4427*), and eleven mRNAs (*ANK1*, *ASTN2*, *COL6A1*, *ITGA2B*, *KCNN3*, *LAMB3*, *LAMC3*, *MST1R*, *RIMS3* and *SLC12A5*). All interaction relationships were encompassed within the network (Figure 10).

### 3.8. Validation of RNA-Seq Results Using qRT-PCR for Laying Traits in Gaoyou Ducks

**Figure 11:**
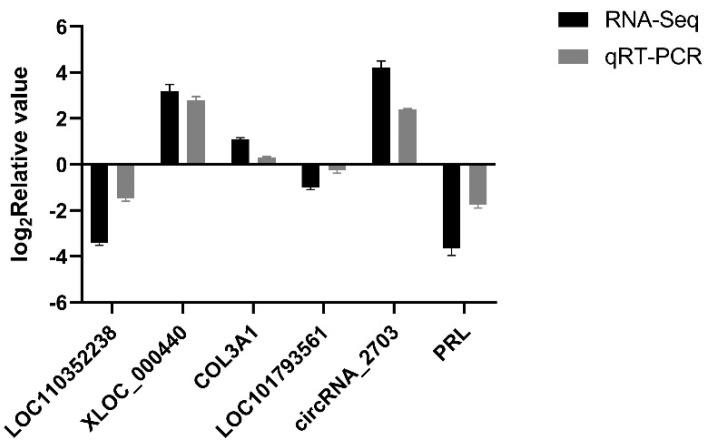
RNA-Seq validation using qRT-PCR. Six transcripts were randomly selected from DEmRNAs, DElncRNAs, and DECircRNAs for qRT-PCR validation to test the accuracy of RNA sequencing.

For qRT-PCR validation (Figure 11), six transcripts were randomly selected from DEmRNAs, DElncRNAs, and DE-CircRNAs. The results showed that some genes, including *XLOC_000440*, *COL3A1*, and *circRNA_2703*, were highly expressed in high-yield Gaoyou ducks’ ovaries, while *LOC110352238*, *LOC101793561*, and *PRL* were highly expressed in low-yield Gaoyou ducks’ ovaries. The comparison between qRT-PCR and RNA-Seq data revealed consistent expression trends for these genes, indicating that the transcriptomic sequencing data and candidate genes from RNA-Seq exhibited high reliability and accuracy. 

## 4. Discussion

In this study, the high-throughput RNA-Seq data comparison between the high- and low-yield egg production traits of Gaoyou ducks’ ovaries revealed 629 DEmRNAs, 279 DElncRNAs, and 62 DEcircRNAS. These results demonstrate that the factors affecting the laying performance of Gaoyou ducks are complex and diverse. To narrow down the potential ceRNAs involved in the egg production of Gaoyou ducks, GO and KEGG enrichment analyses were carried out. This study mainly focused on the specific reproduction GO ontology classification and ovarian steroidogenesis, PI3K-Akt, hedgehog, tryptophan metabolism, and oocyte meiosis signaling pathways, which are involved in follicular development and the selection of egg production performance. Finally, 72 DEmRNAs, 23 DElncRNAs, and 4 DEcircRNAs were obtained. Among the DEmRNAs, some had previously been reported to be involved in the regulation of follicular development or egg performance, including *GDF9* [29,30], *COL1A2* [31], *COL4A1* [31], *COL6A1* [32], *HSD11B2* [33], *DAZL* [23], *PRL* [22], etc. 

The ovarian steroidogenesis signaling pathway regulates hormonal production in ovarian cells and determines ovarian function and ovulation. *GDF9* (growth and differentiation factor 9) [29], a member of the TGF-β family, was significantly enriched in the ovarian steroidogenesis signaling pathway. *GDF9* plays an essential role in ovarian reproductive function. Previous studies have shown that *GDF9* is essential for the folliculogenesis of the primary follicles of mice, and could affect the serum levels of FSH and LH [30]. The PI3K-Akt signaling pathway plays a crucial regulatory role in biological processes such as cell proliferation, differentiation, and apoptosis. Hao et al. [32] compared the granulosa cells’ proteome data of bovine follicles at the dominant follicle and subordinate follicle stages, which demonstrated an association between the PI3K-Akt signaling pathway and follicular development. Bao et al. [33] characterized the RNA-seq profile of ovarian tissue between black Muscovy ducks and white Muscovy ducks with high and low egg production traits; the PI3K-Akt signaling pathway was the most enriched, participating in the follicle development and productivity of Muscovy ducks. Our data show that collagen family genes (*COL1A2*, *COL4A1*, and *COL6A1*), fibroblast growth factors (*FGF5* and *FGF19*), the Laminin subunit family (*LAMB3* and *LAMC3*), and *PRL* were all significantly differentially expressed and enriched in the PI3K-Akt signaling pathway. These findings suggest that these DEGs could be associated with Gaoyou ducks’ ovarian function and affect the specific egg production or double-yolked egg production. 

Numerous studies focusing on different aspects of the lncRNAs, circRNAs, or miRNAs related to the reproductive traits of livestock and poultry have been published in recent years. Furthermore, researchers have been paying more and more attention to the ceRNA regulatory networks, which include coding RNAs, lncRNAs, circRNAs, and miRNAs [34]. Compared to the individual functions of coding and non-coding RNAs, ceRNA regulatory networks connect their functions and offer a singular, all-encompassing viewpoint for illuminating the processes behind the control of biological traits [35,36]. In order to explore the ceRNA regulatory network involved in the egg production trait of Gaoyou ducks, we compared the whole-transcriptome sequencing data between the high- and low-yield egg production traits of Gaoyou ducks’ ovaries. Subsequently, the ceRNA regulatory networks (the circRNA–miRNA–mRNA ceRNA regulatory network and the lncRNA–miRNA–mRNA ceRNA regulatory network) were constructed based on the DEmRNAs, DElncRNAs, DEcircRNAs, and predicted miRNAs of high- and low-yield egg production Gaoyou ducks. Furthermore, four miRNAs (*gga-miR-12273-5p*, *gga-miR-12286-5p*, *gga-miR-1770*, and *tgu-miR-2978*), one lncRNA (*TCONS_00001086*), four circRNAs (*circRNA_1476*, *circRNA_2703*, *circRNA_2754*, and *circRNA_4427*), and eleven mRNAs (*ANK1*, *ASTN2*, *COL6A1*, *ITGA2B*, *KCNN3*, *LAMB3*, *LAMC3*, *MST1R*, *RIMS3*, and *SLC12A5*) were extracted to construct the candidate ceRNA regulatory network. For instance, we found that one specific lncRNA (*TCONS_00001086*) can bind to two different miRNAs (*gga-miR-12273-5p* and *gga-miR-1770*) to regulate the same six genes’ expression levels (*ANK1*, *ASTN2*, *LAMC3*, *MST1R*, *RIMS3*, and *SLC12A5*). One circRNA (*circRNA_1476*) can bind to two different miRNAs (*gga-miR-12273-5p* and *tgu-miR-2978*) to regulate the same seven genes’ expression levels (*ANK1*, *ASTN2*, *COL6A1*, *LAMC3*, *MST1R*, *RIMS3*, and *SLC12A5*). This comprehensive ceRNA regulatory network will be of interest for exploring the regulation mechanism of Gaoyou ducks’ egg production performance. However, due to the limitations of this study, the precise roles of these key DEmRNAs, DElncRNAs, DEcircRNAs, and predicted miRNAs in the egg production process or the double-yolked egg trait of Gaoyou duck need to be validated through further research, both in vivo and in vitro.

## 5. Conclusions

In this study, a comprehensive characterization of key DEmRNAs, DElncRNAs, DEcircRNAs, predicted miRNAs, and signaling pathways associated with egg production performance in Gaoyou ducks was carried out by whole-transcriptome RNA-Seq analysis. The identified ceRNA networks of circRNA/lncRNA–miRNA–mRNA play a vital role in the egg production performance, which offers a novel insight into the regulatory mechanism of ceRNA network in the egg production and double-yolked egg trait of Gaoyou ducks. 

## Data Availability

The data presented in the study are deposited in the Sequence Read Archive in NCBI, accession number PRJNA1033811.

## Supplementary Materials

The following supporting information can be downloaded at: https://www.mdpi.com/article/10.3390/genes15010009/s1, Supplementary Table S1, Primer information for qRT-PCR. Supplementary Table S2, Comparison results of clean data and reference genome.
