# Supplementary material for: Whole-Transcriptome Sequencing of Ovary Reveals the ceRNA Regulation Network in Egg Production of Gaoyou Duck
Source: Genes (Basel). 2023 Dec 20;15(1):9. doi: 10.3390/genes15010009 (PMC10815415; doi:10.3390/genes15010009)
Supplement: Supplementary file 1 [file genes-15-00009-s001.zip › Table S1 Primer information for qRT-PCR.pdf]

Table S1 Primer information for qRT-PCR

Gene	Sequence (5′-3′)	Tm (°C)
LOC110352238	F: GCCCGCCCTTGTCAGTATC<br>R: CCAGGGTCGGTAAGGTTTATTG	58
XLOC_000440	F: GTGGCAGAAATCCATCGTGAA<br>R: CACTGCTGTGTCATGGTTAGC	60
COL3A1	F: CCGTGCCTCCCAGAACATTA<br>R: ACCACAGGCAACCTCATTGT	56
LOC101793561	F: CAGGCTGCCAAGAATGGAGA<br>R: ACGGTGAGTGAAATGCACCT	58
circRNA_2703	F: TGGTGGATCTGGAGGTGTC<br>R: GAGCATCCCTCCACAGCA	60
PRL	F: TGCCTTTTACAACCTGCTGC<br>R: TAGGCGGCACTTCAAAACC	60
GADPH	F：TCGGAGTCAACGGATTTGGC<br>R：TTCTCAGCCTTGACAGTGCC	60
