# Supplementary material for: Whole-Transcriptome Sequencing of Ovary Reveals the ceRNA Regulation Network in Egg Production of Gaoyou Duck
Source: Genes (Basel). 2023 Dec 20;15(1):9. doi: 10.3390/genes15010009 (PMC10815415; doi:10.3390/genes15010009)
Supplement: Supplementary file 1 [file genes-15-00009-s001.zip › Table S2 Clean data statistics for RNA-Seq.pdf]

Table S2 Clean data statistics for RNA-Seq

No.	Sample ID	Total reads	Clean reads	Q30	GC	Mapped ratio	Reads mapped in proper pairs
D1	22	107.67M	105.70M	95.70%	46.21%	90.15%	81.91%
D2	23	102.68M	100.89M	95.85%	46.76%	89.64%	82.09%
D3	24	101.57M	99.69M	95.65%	45.84%	89.99%	82.27%
G1	20	101.32M	99.39M	95.63%	46.89%	89.11%	81.07%
G2	29	106.02M	104.12M	95.79%	46.34%	89.83%	80.59%
G3`	35	109.00M	107.02M	95.79%	46.68%	89.82%	80.99%
